# Pre-treatment functional connectivity of the cingulate cortex predicts anti-suicidal effects of serial ketamine infusions

**DOI:** 10.1192/j.eurpsy.2023.19

**Published:** 2023-03-31

**Authors:** Xiaoyu Chen, Bin Zhang, Shiqi Yuan, Xin Luo, Mingqia Wang, Yiru Hu, Yanling Zhou, Yuping Ning

**Affiliations:** 1 Psychiatric & Psychological Neuroimage Laboratory (PsyNI Lab), The Affiliated Brain Hospital of Guangzhou Medical University, Guangzhou, China; 2 Guangdong Engineering Technology Research Center for Translational Medicine of Mental Disorders, Guangzhou, China; 3 Institute of Mental Health, Tianjin Anding Hospital, Tianjin Medical University, Tianjin, China; 4 Department of Child and Adolescent Psychiatry, The Affiliated Brain Hospital of Guangzhou Medical University, Guangzhou, China; 5 Key Laboratory of Neurogenetics and Channelopathies of Guangdong Province and the Ministry of Education of China, The Second Affiliated Hospital, Guangzhou Medical University, Guangzhou, China.; 6 Department of Psychology, The First School of Clinical Medicine, Southern Medical University, Guangzhou, China

**Keywords:** Cingulate cortex, depression, functional connectivity, ketamine, suicidal ideation

## Abstract

**Background:**

Although ketamine can rapidly decrease suicidal ideation (SI), its neurobiological mechanism of action remains unclear. Several areas of the cingulate cortex have been implicated in SI; therefore, we aimed to explore the neural correlates of the anti-suicidal effect of ketamine with cingulate cortex functional connectivity (FC) in depression.

**Methods:**

Forty patients with unipolar or bipolar depression with SI underwent six infusions of ketamine over 2 weeks. Clinical symptoms and resting-state functional magnetic resonance imaging data were obtained at baseline and on day 13. Remitters were defined as those with complete remission of SI on day 13. Four pairs of cingulate cortex subregions were selected: the subgenual anterior cingulate cortex (sgACC), pregenual anterior cingulate cortex (pgACC), anterior mid-cingulate cortex (aMCC), and posterior mid-cingulate cortex (pMCC), and whole-brain FC for each seed region was calculated.

**Results:**

Compared with non-remitters, remitters exhibited increased FC of the right pgACC–left middle occipital gyrus (MOG) and right aMCC–bilateral postcentral gyrus at baseline. A high area under the curve (0.91) indicated good accuracy of the combination of the above between-group differential FCs as a predictor of anti-suicidal effect. Moreover, the change of SI after ketamine infusion was positively correlated with altered right pgACC–left MOG FC in remitters (*r* = 0.66, *p* = 0.001).

**Conclusions:**

Our findings suggest that the FC of some cingulate cortex subregions can predict the anti-suicidal effect of ketamine and that the anti-suicidal mechanism of action of ketamine may involve alteration of FC between the right pgACC and left MOG.

## Introduction

Suicide is a notably serious public health problem of global dimension, with more than 70,000 people taking their own life annually [1]. The lifetime prevalence of suicidal ideation (SI) is also alarmingly high, estimated at approximately 9.2% according to a cross-national study [2]. Additionally, individuals who present with SI have a higher risk of subsequent suicidal attempts [3, 4]. Compared with the general population, patients with depression are more likely to suffer from SI, and as SI is an important prodrome of suicidal attempts and suicide, there is a burgeoning interest in its prevention [5, 6]. However, commencement of lithium and clozapine may take weeks to exert its anti-suicidal effects [7]. Therefore, there has been a dearth of acute treatment for SI.

Ketamine is a noncompetitive N-methyl-d-aspartate (NMDA) receptor antagonist that has recently been indicated as having the capacity to reduce SI rapidly in both major depressive disorder (MDD) and bipolar disorder (BD) patients [8–10]. A meta-analysis has shown the promising effects of a single dose of ketamine on SI as an emergency treatment, with the effects being apparent for up to 1 week [11]. Moreover, Phillips et al. [12] have reported that repeated ketamine infusions can elicit accumulating reductions in SI score in patients with depression, indicating a prospective intervention for SI. However, although the anti-suicidal effects of ketamine appear to be impressive, its precise mechanism of action is unclear.

The cingulate cortex, which surrounds the corpus callosum, has been identified as a critical hub for affect and motivation [13]. Based on the function and anatomical structure of the cingulate cortex, its two most anterior subregions are referred to as the anterior cingulate cortex (ACC) and mid-cingulate cortex (MCC) [13]. The ACC is further subdivided into the subgenual ACC (sgACC) and pregenual ACC (pgACC) in consideration of distinct patterns of connectivity, whereas the MCC, which is located in the middle third of the cingulate cortex, can be further divided into the anterior MCC (aMCC) and posterior MCC (pMCC) [13]. Recently, emerging neuroimaging studies have demonstrated that structural [14], functional [15], and metabolic [16, 17] alterations in the ACC are associated with suicide. Notably, SI is linked with dysfunctional emotional processes, and as the ACC is the affect processing center [16], this suggests that it may have a role in SI from the perspective of neural mechanism. In one study, the bilateral sgACC has found to be hypo-active in patients with depression who attempted suicide [18]. Moreover, it has been established that the ACC is a critical locus of ketamine action and that the activity of the sgACC and pgACC can be rapidly altered by ketamine treatment [19]. Considerable studies have concluded that the pre-treatment activity of the pgACC is associated with the antidepressant effect of ketamine [20, 21]. However, neuroimaging literature regarding the pgACC has predominately focused on individuals who suffer from depression but who have current SI. Upregulation of the functional connectivity (FC) between the MCC (often referred to in the literature as “dorsal ACC”) and ACC regions is related to the anti-suicidal effect of ketamine [13, 22]. Additionally, dorsal ACC hyperconnectivity with the dorsal fronto-parietal cortex as well as relative hypoconnectivity with the bilateral ventrolateral prefrontal cortex is related to a history of SI [23], implying that changes in functional interactions between different MCC subregions are likely to be distinct. The activity of the aMCC is linked to negative affect experience [24]. In terms of receptor-related architecture, the aMCC has recently been reported to have a high density of NMDA receptors [25], which bolsters the theory that ketamine exerts its effect on the aMCC. Nevertheless, very few studies have explored the association between the pMCC and the effects of ketamine in detail.

To the best of our knowledge, the FC between ACC and MCC subregions has not been directly compared from the perspective of association with the anti-suicidal effect of ketamine. As insights into the neuropharmacology of ketamine will facilitate the development of suicide prevention strategies, in the present study, we employed seed-based FC analysis and receiver operating characteristic (ROC) curves analysis to determine (a) whether SI remitters showed different FC within the ACC and MCC subregions compared with non-remitters; (b) whether the differential FC between remitters and non-remitters could emerge as predictive biomarkers of treatment outcomes; and (c) whether alterations in FC were related to improvements in SI-related symptoms. We hypothesized that FCs of the ACC or MCC subregions could represent predictive biomarkers for SI remission and that their alterations might be linked to the anti-suicidal effect of ketamine.

## Methods

### Participants

The study was approved by the local Ethics Committee of the Affiliated Brain Hospital of Guangzhou Medical University and was part of an open-label clinical trial with regard to repeated ketamine infusion treatment for subjects with depression. The trial was registered in the Chinese Clinical Trial Registry, numbered ChiCTR-OOC-17012239. All participants provided written informed consent prior to enrolment in the trial.

Participants were recruited from the Affiliated Brain Hospital of Guangzhou Medical University between September 2016 and December 2017. Subjects were eligible for the study if they (a) were between 18 and 65 years of age, regardless of sex; (b) met the structured clinical interview for the fifth edition of the Diagnostic and Statistical Manual Disorders (DSM-V) criteria for MDD or BD without psychotic features; (c) experienced SI, defined as a Beck Scale for Suicide Ideation (SSI)-part I score of ≥2 [26]; (d) scored ≥17 on the 17-item Hamilton Depression Rating Scale (HAMD-17) at baseline; and (e) had baseline resting-state functional magnetic resonance imaging (rs-fMRI) data. The detailed exclusion criteria have been exposited previously [27].

### Study design

Forty participants received six sub-anesthetic doses of ketamine (0.5 mg/kg over 40 min) via intravenous infusions for 12 days (days 1, 3, 5, 8, 10, and 12). More details regarding the study design have been described in our previous studies [28, 29]. All patients were on antidepressant medications at stable dosage 4 weeks prior to screening and throughout the study. Clinical symptoms were assessed at baseline (24 h prior to infusion) and on day 13 (24 h following the sixth infusion), and MRI scans were acquired at the same time points. All participants were required to complete the HAMD-17 questionnaire for the assessment of severity of depressive symptoms [30]. Additionally, the Beck SSI-part I was applied to reflect the severity of SI [31]. Participants were considered to be in complete remission if their score on day 13 was zero, and those who scored more than zero were defined as non-remitters.

### MRI data acquisition

All rs-fMRI scans were operated on a 3.0 T MRI scanner (Achieva X-series, Philips Medical Systems, Best, the Netherlands). Imaging data were acquired for an 8-min resting-state sequence using gradient echo planar imaging (EPI) with the following parameters: repetition time (TR) = 2,000 ms, echo time (TE) = 30 ms, field of view (FOV) = 220 × 220 mm^2^, voxel size = 3.44 × 3.44 × 4 mm^3^, matrix size = 64 × 64, number of slices = 33, slice thickness = 4 mm, and slices gaps = 0.6 mm. During the scanning process, all participants were requested to remain awake with their eyes closed.

### Image processing

Imaging data pre-processing was carried out utilizing Data Processing & Analysis for Brain Imaging (DPABI, V5.0, http://www.rfmri.org/dpabi) in MATLAB R2014a software (The Mathworks, Inc., Natick, MA). The first 10 images were removed, and the residual fMRI images were corrected for slice time and head motion. To exclude the effect of head motion, the Friston 24-parameter model was conducted [32]. Nuisance covariates, including white matter and cerebrospinal fluid, were regressed out in a general linear model, but the global signal remained [33]. All imaging data were band-pass filtered (0.01 Hz < *f* < 0.1 Hz). All filtered images were resliced to 3 × 3 × 3 mm^3^ and normalized to the Montreal Neurological Institute (MNI) space with EPI template. The normalized images were smoothed using a 6-mm full-width-half-maximum Gaussian kernel. Participants whose heads moved excessively (>3 mm of displacement or >3° of rotation in any direction) and whose normalized images failed to match well with the EPI template were excluded. Two remitters and two non-remitters were excluded from the FC analysis because of poor quality of pre-treatment images. Consequently, 40 participants were finally enrolled in the study.

### FC analysis

Consistent with the previous study by Davey et al. [34], we selected eight spherical seed regions of interest (ROIs) with a radius of 3.5 mm centered on eight MNI coordinates: the subgenual ACC [MNI (*x*, *y*, *z*): left = −5, 25, −10; right = 5, 25, −10], the pregenual ACC [MNI (*x*, *y*, *z*): left = −5, 38, 6; right = 5, 38, 6], the anterior MCC [MNI (*x*, *y*, *z*): left = −5, 27, 21; right = 5, 27, 21], and the posterior MCC [MNI (*x*, *y*, *z*): left = −5, 10, 33; right = 5, 10, 33]. For each subject, seed-based FC analysis between these eight ROIs and the whole brain was conducted on the Resting-State fMRI Data Analysis Toolkit plus V1.22 (RESTplus, V1.22, http://restfmri.net/forum/). Pearson’s correlation coefficients between mean blood-oxygenation-level-dependent time course values extracted from each ROI and the time course values of all other voxels in the whole brain were calculated. These coefficients were then converted to *Z*-values by means of Fisher’s *r*-to-*z* transformation to obtain subject-level FC maps.

### Statistical analysis

Patients who had received all six intravenous doses of ketamine and for whom functional images at baseline were available were included in the final analysis; 20% (8/40) of patients lacked post-treatment functional images. The two-sample *t*-test, chi-square test, and Mann–Whitney *U*-test were used to compare the demographic and clinical characteristics between remitters and non-remitters using the Statistical Package for Social Sciences version 23 (IBM, Chicago, IL).

The two-sample *t*-test was performed to assess the between-group pre-treatment differential FC of the whole brain with each ROIs on SPM12 toolbox (https://www.fil.ion.ucl.ac.uk/spm). Age, age of onset, and SSI-part I score were included as nuisance covariates. The cluster-level threshold was set at *p* < 0.05, with false discovery rate (FDR) correction, and the voxel-wise threshold was set at *p* < 0.001. To determine whether significant pre-treatment between-group differences in FC were related to the change in depressive and SI symptoms in remitters, Pearson correlation analysis was performed to calculate the associations between pre-treatment differential FC and changes in total HAMD-17 and SSI-part I scores.

The paired *t*-test was conducted, respectively, in remitters and non-remitters using the pre-treatment and post-treatment image data, with pre- and post-treatment SSI-part I score as the covariates. The cluster-level threshold was set at *p* < 0.05, with FDR correction, and the voxel-wise threshold was set at *p* < 0.001. The correlations between significantly altered FC and changes in total HAMD-17 and SSI-part I scores were assessed in remitters.

In addition, ROC curves were plotted to evaluate the predictive value of baseline between-group differences in FC for the anti-suicidal effect of ketamine. Optimal cut-off points were acquired from the Youden index (maximum [sensitivity + specificity − 1]), and area under the curve (AUC), 95% confidence intervals (CIs), sensitivity, and specificity values were calculated for each optimal cut-off point. Statistical significance was set at *p* < 0.05.

## Results

### Participants characteristics

Twenty-five patients (62.5%) were classified as remitters and 15 (37.5%) as non-remitters. The remitters manifested significantly lower SSI-part I score (*t* = −2.622, *p* = 0.013). Compared with non-remitters, remitters differed significantly in age (*t* = 4.010, *p* < 0.001) and age of onset (*Z* = −2.607, *p* = 0.009). There were no significant differences between remitters and non-remitters in terms of sex, HAMD-17 total scores, diagnosis (MDD vs. BD), or duration of illness. Further details are provided in Table 1.

**Table 1.** Baseline demographic and clinical characteristics of the participants.

	Total sample		Remitter		Non-remitter		Statistics	
	(*n* = 40)		(*n* = 25)		(*n* = 15)			
	*n*	%	*n*	%	*n*	%	*χ* ^2^	*p*
Gender(female)	21	52.5	13	52.0	8	53.3	0.007^a^	0.935
Diagnosis (MDD)	32	80.0	22	88.0	10	66.7	1.500^b^	0.221
History of psychiatric hospitalization	11	27.5	6	24.0	5	33.3	0.075^b^	0.784
Family history of psychiatric disorders	15	37.5	11	44.0	4	26.7	1.202^a^	0.273
Current antidepressant medications								
SSRIs	1	2.5	1	4.0	0	0.0	0.000^b^	1.000
SNRIs	1	2.5	0	0.0	1	6.7	0.068^b^	0.794
NDRIs	1	2.5	1	4.0	0	0.0	0.000^b^	1.000
NaSSAs	3	7.5	2	8.0	1	6.7	0.000^b^	1.000

	Mean	SD	Mean	SD	Mean	SD	*t*/*Z*	p
Age (years)	32.85	11.36	37.20	11.36	25.60	6.94	4.010^c^	**<0.001**
Education (years)	12.18	3.15	12.12	3.50	12.27	2.55	−0.198^d^	0.843
BMI (kg/m^2^)	22.98	3.38	23.25	3.37	22.54	3.48	0.638^c^	0.528
Age of onset (years)	25.28	10.75	28.72	11.74	19.53	5.42	−2.607^d^	**0.009**
Duration of illness (months)	88.57	78.59	99.20	92.90	70.87	43.30	−0.266^d^	0.790
HAMD-17 total score	24.00	4.66	23.28	3.89	25.20	5.67	−1.271^c^	0.212
SSI-part I score	5.53	2.69	4.72	2.32	6.87	2.80	−2.622^c^	**0.013**

*Note*: Values are marked in bold if *p* < 0.05.

Abbreviations: BMI, body mass index; HAMD-17: the 17-item Hamilton Rating Scale for Depression; MDD, major depressive disorder; NaSSAs, noradrenergic and specific serotonergic antidepressants; NDRIs, norepinephrine-dopamine reuptake inhibitors; SNRIs, serotonin and norepinephrine reuptake inhibitors; SSI-part I, Beck Scale for Suicide Ideation part I; SSRIs, selective serotonin reuptake inhibitors.

a Chi-square test.

b Continuity correction chi-square test.

c Two-sample *t*-test.

d Mann–Whitney *U*-test.

### Pre-treatment neuroimaging predictors of treatment outcome

**Figure 1.**
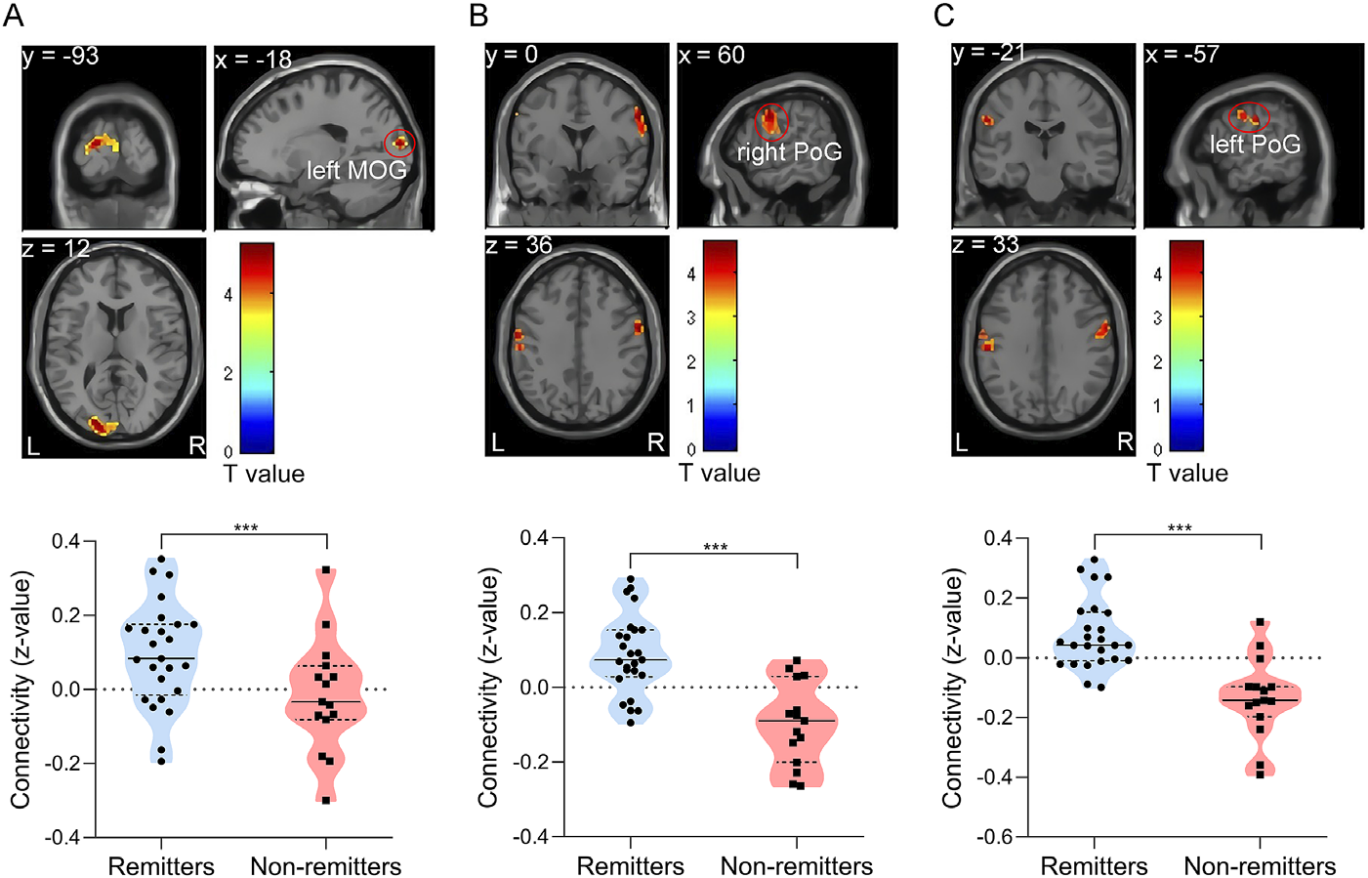
Significantly differential FC at baseline between remitters and non-remitters (*p* < 0.05, FDR cluster-level corrected). (A) Remitters had stronger FC between the right pgACC and the left MOG. (B) Remitters exhibited stronger FC between the right aMCC and the right PoG. (C) Remitters exhibited stronger FC between the right aMCC and the left PoG. The color bar represents the two-sample *t*-test *t-*values. *Abbreviations*: ***, significant after FDR correction for multiple comparisons; aMCC, anterior mid-cingulate cortex; FC, functional connectivity; FDR, false discovery rate; L (R), left (right) hemisphere; MOG, middle occipital gyrus; PoG, postcentral gyrus; pgACC, pregenual anterior cingulate cortex.

The two-sample *t*-test revealed that right pgACC-based FC showed a significant left middle occipital gyrus (MOG) cluster (*p* < 0.05, FDR cluster-level corrected, peak voxel (*x*, *y*, *z*) = (−18, −93, 12), cluster = 103, *T* = 5.23). Specifically, remitters had stronger FC between the right pgACC and left MOG than non-remitters. Remitters also exhibited enhanced FC between the right aMCC and the right postcentral gyrus (PoG) (*p* < 0.05, FDR cluster-level corrected, peak voxel (*x*, *y*, *z*) = (60, 0, 36), cluster = 101, *T* = 4.71) and the left PoG (*p* < 0.05, FDR cluster-level corrected, peak voxel (*x*, *y*, *z*) = (−57, −21, 33), cluster = 63, *T* = 4.43) (Table 2 and Figure 1). No significant correlations were identified between the change in HAMD-17 and SSI-part I scores, and pre-treatment right pgACC–left MOG, right aMCC–right PoG, and right aMCC–left PoG connectivity in remitters (Supplementary Figure S1).

**Table 2.** The areas of significantly differential FC between the remitters and the non-remitters.

Seeds	Location in the cerebrum	MNI coordinates			*T*-value	Voxels	*p*
		*x*	*y*	*z*			
R pregenual anterior cingulate cortex	L middle occipital gyrus	−18	−93	12	5.226	103	0.0165
R anterior mid-cingulate cortex	R postcentral gyrus	60	0	36	4.711	101	0.0118
	L postcentral gyrus	−57	−21	33	4.426	63	0.0438

*Note*: *p*-value was corrected by false discovery rate.

Abbreviation: MNI, Montreal Neurological Institute.

**Figure 2.**
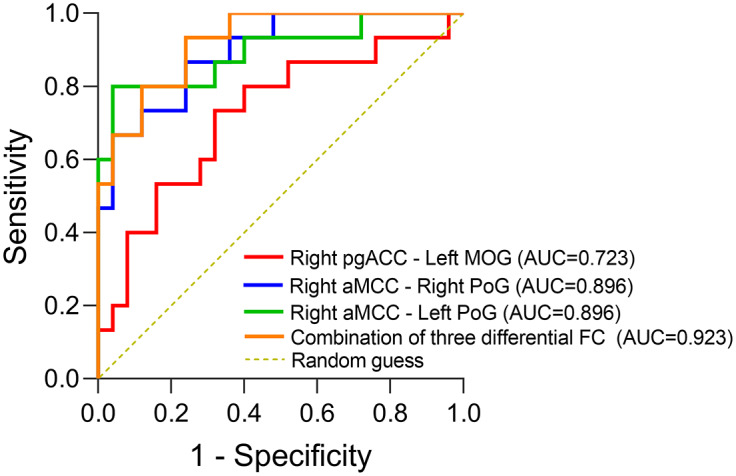
ROC curves for the classification of remission status with pre-treatment differential connectivity between groups. *Abbreviations*: AUC, area under curve; ROC, receiver operating characteristic.

Regarding classification of remitters and non-remitters, ROC curve analysis indicated that right pgACC–left MOG, right aMCC–right PoG, and right aMCC–left PoG connectivities were significant predictors of an acute anti-suicidal effect on day 13. The AUC of the right pgACC–left MOG connectivity was 0.72 (95% CI, 0.55–0.89; *p* < 0.05), the AUC of the right aMCC–right PoG connectivity was 0.90 (95% CI, 0.80–0.99; *p* < 0.001), and the AUC of the right aMCC–left PoG connectivity was 0.90 (95% CI, 0.78–1.00; *p <* 0.001). Moreover, the highest AUC (0.92) was found for the combination of these three predictors (95% CI, 0.84–1.00; *p* < 0.001). The ROC curves of the detection rendered from the SI remission are shown in Figure 2, and the Youden index, optimal cut-off point, specificity, and sensitivity values are provided in Supplementary Table S1.

### Association between clinical efficacy and FC changes

**Figure 3.**
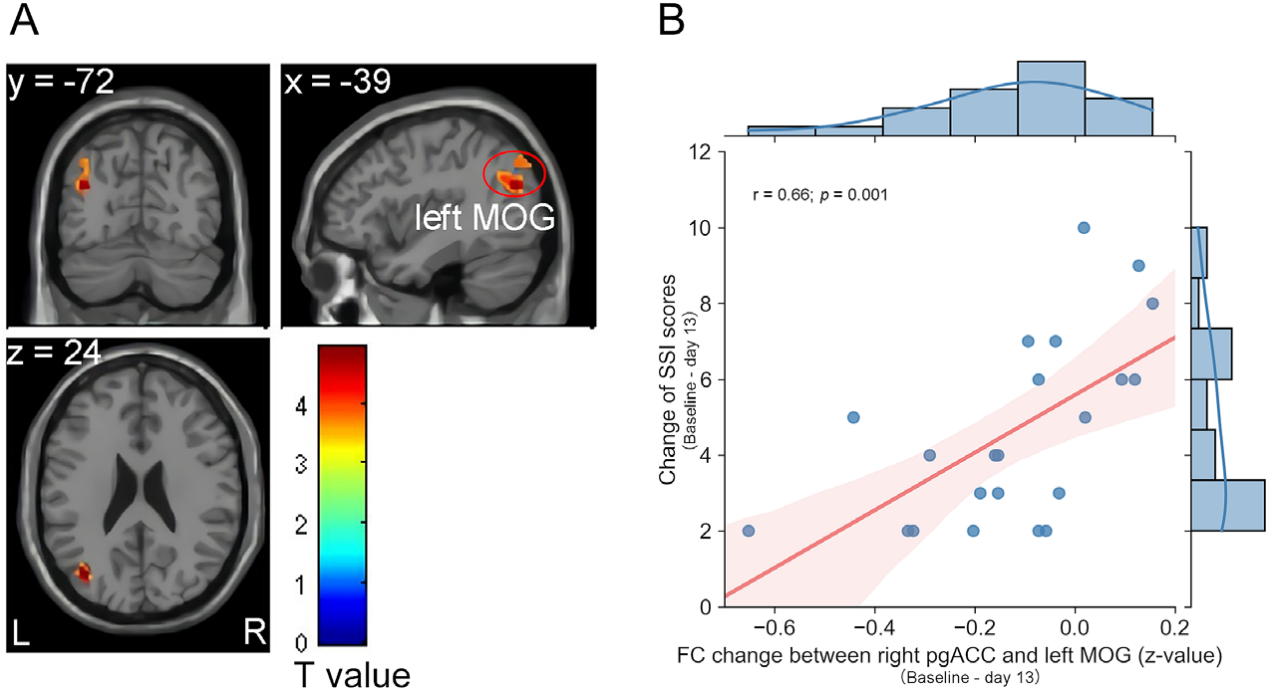
Altered FC was correlated with the anti-suicidal effects of ketamine. (A) Remitters had enhanced FC between the right pgACC and the left MOG after repeated ketamine infusions (*p* < 0.05, FDR cluster-level corrected). (B) Altered FC between the right pgACC and the left MOG was positively related to the change of SI (*r* = 0.66, *p* = 0.001). The color bar represents the pair *t*-test *t*-values. *Abbreviations*: FC, functional connectivity; FDR, false discovery rate; L (R), left (right) hemisphere; MOG, middle occipital gyrus; pgACC, pregenual anterior cingulate cortex; SSI, Beck Scale for Suicide Ideation (SSI)-part I.

Remitters showed a significant increase in right pgACC–left MOG FC (*p* < 0.05, FDR cluster-level corrected, peak voxel (*x*, *y*, *z*) = (−39, −72, 24), cluster = 70, *T* = 4.95) (Figure 3A), whereas non-remitters showed increased left pMCC–right lateral ventricle FC (*p* < 0.05, FDR cluster-level corrected, peak voxel (*x*, *y*, *z*) = (12, −24, 18), cluster = 69, *T* = 11.85). Significantly altered FC between the right pgACC and the left MOG was positively correlated with the change of SSI-part I score in remitters (Figure 3B; *r* = 0.66, *p* = 0.001), whereas there was no such association with change in HAMD-17 score (*r* = −0.08, *p* > 0.05). The right aMCC–bilateral PoG FC was not significantly altered in remitters, and the altered right aMCC–bilateral PoG FC in individual patients was not significantly associated with the change of SSI-part I and HAMD-17 scores.

## Discussion

The present study found that remitters exhibited pre-treatment hyperconnectivity between the right pgACC and the left MOG and between the right aMCC and the bilateral PoG compared with non-remitters. Furthermore, differential pre-treatment FC predicted SI remission after ketamine treatment. Among the remitters, altered connectivity after ketamine treatment between the right pgACC and the left MOG was positively related to the change of SI. To the best of our knowledge, this is the first study to examine the altered FC within ACC and MCC subregions in patients with depression and SI and to explore the related FC in terms of biomarkers for SI remission.

Prior studies have indicated that the pgACC has a pivotal role in depression; most notably, pre-treatment activity of pgACC was found to predict subsequent antidepressant response to several treatment modalities (repetitive transcranial magnetic stimulation [35] and medicine [20, 36, 37]). The current study provided an account for the role of the pgACC in SI remission in depression, as pre-treatment right pgACC–left MOG connectivity was a putative biomarker of remission. In addition, we found that ketamine up-regulated right pgACC–left MOG connectivity in remitters and that its alteration was associated with the anti-suicidal effect of ketamine. A previous study noted that FC between the pgACC and the superior frontal gyrus was correlated with the severity of SI [37]. Notably, the pgACC plays a critical role in the antidepressant action of ketamine [19]. In parallel, ketamine has been found to induce a rapid increase in FC between the pgACC and the right caudate nucleus, and its alteration has been linked to improvement in depressive symptoms [38]. Taken together, these results suggest that the pgACC may also be a crucial hub of anti-suicidal action of ketamine. Albeit aMCC–ventral striatum connectivity has been proved to predict depressed mood [39], there are no studies pertaining to aMCC connectivity and its association with SI. The aMCC has been distinguished as a critical region for cognitive control and negative affect [24]. Deficient cognitive control has been linked with suicidality, as those who have SI or attempt suicide, perform poorer cognitive control than healthy controls [40]. Moreover, aspects of negative affect, such as hopelessness, worthlessness, and low self-esteem, emerge as risk factors for the future SI and suicidal behaviors [41]. Our results showed that pre-treatment FC between the right aMCC and the bilateral PoG could be a predictive biomarker of SI remission in depression. Moreover, quinolinic acid (QUIN) concentration has been shown to increase specifically in the aMCC of suicidal MDD patients [42]. QUIN is an NMDA receptor agonist, and as the NMDA receptors are rich in aMCC, the connectivity between these regions at baseline might reflect the glutamatergic dysfunction in non-remitters, with the underlying glutamatergic function in turn predicting the subsequent treatment outcome. The present study extends these findings with regard to aMCC and SI, and strengthens the role of aMCC as a target of treatment. In other words, the pgACC and the aMCC might be pivotal brain regions in depressed patients with SI who eventually achieved complete SI remission with ketamine treatment.

The MOG, as part of the visual cortex areas, plays a vital role in processing emotional stimuli [43]. Prior research has shown that neural activity of the bilateral middle occipital cortex during the processing of emotional information can be a biomarker for subsequent antidepressant response [44]. Moreover, a coordinate‑based meta‑analysis demonstrated hyperactivity in the right MOG in SI patients relative to controls [45]. Similarly, highly suicidal depressed youth show higher activity in the MOG than do low suicidal depression and healthy controls [46]. Likewise, zALFF in the left MOG is a putative predictor of depression with and without suicidal attempts [43]. Importantly, our results indicated that the anti-suicidal effect of ketamine was predicted by pre-treatment FC between the right pgACC and the left MOG, thus extending the role of the MOG in predicting treatment outcome. Our results also implied that there might be the ACC–visual cortex circuitry impacting SI. Nevertheless, no studies have yet proposed the ACC–visual cortex circuitry, and hopefully there will be further research on this circuitry.

Regarding the PoG, our current findings indicated pre-treatment hyperconnectivity between the right aMCC and the bilateral PoG in remitters. The PoG, which pertains to the somatosensory cortex, plays a critical role in impulse control and emotional regulation [47], and has been implicated in SI [48]. MDD patients with SI show decreased dynamic FC from the habenula to the PoG, suggesting that less frequent switching of FC between the two regions is linked with abnormal emotional response [49]. A possible interpretation of our results is that higher FC between the right aMCC and the bilateral PoG may contribute to the function of emotional regulation, leading to SI remission. The association suggested herein between the aMCC and the PoG may provide insights into the underlying nature of predictors of SI remission.

Nevertheless, the small sample size can be considered a possible limitation of the current study. Although 40 participants were enrolled in the study, eventually 32 individuals were included in the advance analysis because of the absence of post-treatment imaging data. Additionally, participants received the ketamine infusions with concurrent antidepressant medications, which might affect the observed response to ketamine. However, the add-on ketamine study was more appropriate for severe depression, and it could be conducive to providing real-world data [50].

Our findings may provide the foundation for individualized clinical therapy based on utilization of FC biomarkers for recognizing the potential SI remitters. From the perspective of mechanism, our results suggest that longitudinal pgACC and MOG connectivity contributes to the understanding of the neural mechanism underlying the anti-suicidal effect of ketamine.

## Data Availability

The data included in this study are available from the corresponding author upon reasonable request.

## Supplementary material

For supplementary material accompanying this paper visit http://doi.org/10.1192/j.eurpsy.2023.19. click here to view supplementary material
