# Supplementary figures and images for: Pre-treatment functional connectivity of the cingulate cortex predicts anti-suicidal effects of serial ketamine infusions
Source: Eur Psychiatry. 2023 Mar 31;66(1):e31. doi: 10.1192/j.eurpsy.2023.19 (PMC10134448; doi:10.1192/j.eurpsy.2023.19)

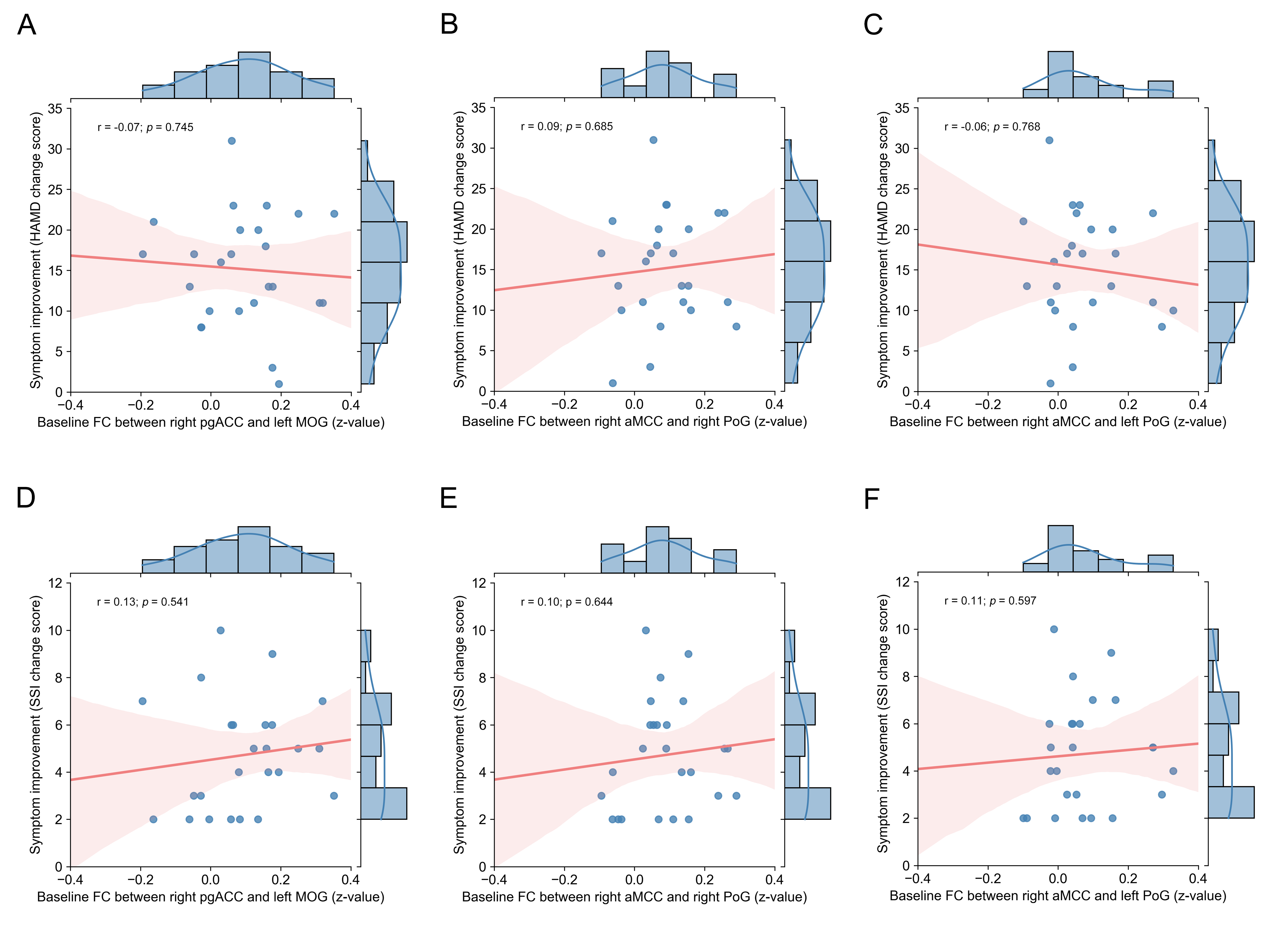

Supplement: Supplementary file 1 [file epasup.zip › S0924933823000196sup001.tif]
